# Development and Validation of a Phenotyping Computational Workflow to Predict the Biomass Yield of a Large Perennial Ryegrass Breeding Field Trial

**DOI:** 10.3389/fpls.2020.00689

**Published:** 2020-05-28

**Authors:** Alem Gebremedhin, Pieter Badenhorst, Junping Wang, Fan Shi, Ed Breen, Khageswor Giri, German C. Spangenberg, Kevin Smith

**Affiliations:** ^1^Agriculture Victoria, Hamilton Centre, Hamilton, VIC, Australia; ^2^Faculty of Veterinary and Agricultural Sciences, School of Agriculture and Food, The University of Melbourne, Melbourne, VIC, Australia; ^3^Agriculture Victoria, AgriBio, Centre for AgriBioscience, Bundoora, VIC, Australia; ^4^School of Applied Systems Biology, La Trobe University, Bundoora, VIC, Australia

**Keywords:** high-throughput phenotyping, biomass, perennial ryegrass, NDVI, plant height, computational workflow

## Abstract

Increasing dry matter yield (DMY) is the most important objective in perennial ryegrass breeding programs. Current yield assessment methods like cutting are time-consuming and destructive, non-destructive measures such as scoring yield on single plants by visual inspection may be subjective. These assessments involve multiple measurements and selection procedures across seasons and years to evaluate biomass yield repeatedly. This contributes to the slow process of new cultivar development and commercialisation. This study developed and validated a computational phenotyping workflow for image acquisition, processing and analysis of spaced planted ryegrass and investigated sensor-based DMY yield estimation of individual plants through normalized difference vegetative index (NDVI) and ultrasonic plant height data extraction. The DMY of 48,000 individual plants representing 50 advanced breeding lines and commercial cultivars was accurately estimated at multiple harvests across the growing season. NDVI, plant height and predicted DMY obtained from aerial and ground-based sensors illustrated the variation within and between cultivars across different seasons. Combining NDVI and plant height of individual plants was a robust method to enable high-throughput phenotyping of biomass yield in ryegrass breeding. Similarly, the plot-level model indicated good to high-coefficients of determination (*R*^2^) between the predicted and measured DMY across three seasons with *R*^2^ between 0.19 and 0.81 and root mean square errors (RMSE) values ranging from 0.09 to 0.21 kg/plot. The model was further validated using a combined regression of the three seasons harvests. This study further sets a foundation for the application of sensor technologies combined with genomic studies that lead to greater rates of genetic gain in perennial ryegrass biomass yield.

## Introduction

Increasing biomass yield is the most important trait to improve during the breeding of perennial ryegrass (Smith et al., 2001; McDonagh et al., 2016; Herridge et al., 2018; Ghamkhar et al., 2019). However, biomass yield is a complex trait, which varies with the number and density of tillers, regrowth after defoliation and growth habit; across a range of seasons, environments, and plant age (Yates et al., 2019). Furthermore, the early stages of perennial ryegrass breeding programs depend on the assessment of populations based on large numbers of genotypes planted as spaced plants or small sward plots in the field (Lootens et al., 2016; Ghamkhar et al., 2019). These assessments involve multiple measurement and selection procedures across seasons and years to repeatedly evaluate biomass yield (Leddin et al., 2018). Biomass yield assessment methods are time-consuming, mainly relying on visual scores or destructive harvesting, drying and weighing of samples. This contributes to the slow process of new cultivar development and commercialization. The application of sensor-based high-throughput phenotyping (HTP) technologies on aerial and ground-based mobile platforms have the potential to offer a non-destructive, rapid and efficient method to assess biomass yield in the field (Inostroza et al., 2016; Gebremedhin et al., 2019b; Ghamkhar et al., 2019).

The possibility for the application of HTP for automated biomass assessments on an objective and accurate basis has been demonstrated for forage grasses (Walter et al., 2012; Barrett et al., 2015). In other species, various remote sensing methods have been calibrated and validated to conduct measurements at a single plant, plot or experimental units, paddock and landscape scales (Rahman et al., 2014; Hunt et al., 2015; Inostroza et al., 2016; Punalekar et al., 2018). Satellite-based remote sensing methods have been used to assess grassland biomass at a landscape scale (Barrachina et al., 2015). However, low temporal and spatial resolution limits the applicability of satellite remote sensing for breeding (Tattaris et al., 2016). For instance, satellite imaging tools have spatial resolution ranging from 1.24 to 260 m (Chawade et al., 2019), which limits their application on small experimental plots. Proximal sensing platforms have the advantage of capturing phenotypic data from thousands of genotypes at high spatial and temporal resolution (Haghighattalab et al., 2016). The platforms equipped with various sensors may function to capture information of multiple traits from field plots and individual plants.

A variety of aerial and ground-based HTP platforms have been developed for non-destructive biomass estimation of wheat (Yue et al., 2017; Jimenez-Berni et al., 2018), barley (Bendig et al., 2014; Brocks and Bareth, 2018), triticale (Busemeyer et al., 2013), and rice (Devia et al., 2019). Specifically, small grassland experiments (10 sampled plots of 1 m^2^ each), demonstrated non-destructive biomass estimations of timothy and ryegrass from plant height (Rueda-Ayala et al., 2019). Other experiments of biomass estimation on ryegrass (Borra-Serrano et al., 2019) and a mixture of timothy and meadow fescue (Viljanen et al., 2018) were demonstrated using aerial platforms. In these studies, vegetative indices, plant height and photogrammetric features were validated, and the models from these surrogates were used to develop biomass estimation models under plots and small paddock level (*n* ≤ 96 sampled plots) experiments. Thus, the general application of aerial and ground-based phenotyping methods in these experiments showed there is a potential to increase throughput and hence to improve the precision to individual plants level biomass yield estimation. Therefore, development and validation of a phenotyping computational workflow of data acquisition, processing and analysis could be applied to implement automated biomass yield estimation in large-scale ryegrass breeding program. We have recently shown that normalized difference vegetative index (NDVI) and plant height correlated with perennial ryegrass biomass yield in four seasons (Gebremedhin et al., 2019a). The results showed combining NDVI and plant height to be a robust method to enable high-throughput phenotyping of biomass yield in a ryegrass breeding program.

The objective of the study was to validate a computational phenotyping workflow for image acquisition, processing and analysis of spaced-planted perennial ryegrass to estimate the biomass yield of 48,000 individual plants through NDVI and plant height data extraction. The workflow resulted in the accurate, non-destructive biomass yield estimation of spaced planted perennial ryegrass across growing seasons in a field environment. The outcome of the study contributes to the application of sensor technologies combined with genomic selection for greater rates of genetic gain in forages.

## Materials and Methods

### Field Experiment and Plant Material

Advanced perennial ryegrass breeding lines and commercial cultivars were established in a field experiment in June 2016 at Agriculture Victoria Research, Hamilton Centre, Victoria, Australia (37.8464°S, 142.0737°E) (Supplementary Figure 1), as part of a project to implement genomic selection (GS) in perennial ryegrass. Fifty advanced perennial ryegrass breeding lines and commercial cultivars were planted in a randomized complete block design with ten replications of each. Each line or commercial cultivar was coded 1–50 to conceal their identities as requested by the owners of the breeding lines and cultivars. Each replication was considered as a plot and contained three rows of 32 spaced plants each (i.e., 96 plants/plot). The experimental unit was, therefore, a plot of 8 × 1.8 m. The spacing between plants was 25 cm and between rows was 60 cm. The GS experiment contained a total of 48,000 individual plants in 10 blocks. The total area of the field experiment was 8,100 m^2^.

### Phenotypic Data Collection

For the non-destructive biomass estimation, ground and aerial-based proximally sensed data were collected a day before each harvest. The harvests were allocated to seasons according to the protocol of Leddin et al. (2018). The field experiment was harvested three times in 2018, one in June 2018 called winter 2018, and two in the late spring season of October 2018 and November 2018 called late-spring2018_1 and late-spring2018_2, respectively. No harvests were conducted in autumn or summer due to slow growth and drought. In these three harvests, two sets of data collection scenarios were conducted at the same time.

Firstly, 480 individual plants from three breeding lines were selected and manually harvested at 5 cm above ground level in each of the seasonal harvests. The manually harvested measurements were used to develop and validate dry matter yield (DMY) estimation model from the combinations of NDVI and plant height values from sensors (Gebremedhin et al., 2019a). The models developed from these individual plants were used to estimate biomass of the 48,000 plants for the winter2018, spring2018_1, and late-spring2018_2 seasonal harvests.

Secondly, mechanical harvesting of the 500 plots of the GS field was conducted using a Gianni Ferrari combine harvester (Gianni Ferrari s.r.l., Reggiolo, Italy) to validate the plot level correlation between estimated and measured DMY. In the late-spring2018_1 and late-spring2018_2 measured DMY data wasn’t collected from 100 plots since these plots were allocated for flowering score experiment. Plant height data from a few plots of the late-spring2018_2 was not received due to technical issues of the Phenorover driving at that specific date.

### Aerial Images Acquisition and Processing

Multispectral images acquisition was conducted using a RedEdge-M sensor (RedEdge, MicaSense Inc., Seattle, WA, United States) attached to DJI M100 quadcopter (DJI Technology Co., Shenzhen, China). The RedEdge-M capture images simultaneously at five bands including blue (465–485 nm), green (550–570 nm), red (663–673 nm), red edge (712–722 nm), and near-infrared (820–860 nm). It also has GPS and sensor and incident light sensors. The flight mission was planned by Pix4D Capture software.

Aerial images were collected using the unmanned aerial system (UAS) on a weekly basis over the GS trial site, and three sets of data from 2018 (winter2018, late-spring2018_1, and late-spring2018_2) was used for this analysis. Imaging dates were synchronized with each harvest. Flight operations were conducted under bright, sunny weather conditions to minimize noise from environmental variation. The UAS flight altitude was set at 20 m above ground level, and the flight speed was 6 m/s with 75% side and forward overlap of images. At this flight altitude and speed, the spatial resolution of the images was 2 cm/pixel. The same flight path was followed on each date. Multispectral ground calibration targets (Tetracam Inc., Chatsworth, CA, United States) with known reflectance values (6, 11, 22, and 33%) was used for radiometric calibration by developing regression equation between digital numbers (DN) vs. the reflectance percentage.

### Computational Workflow for Data Extraction

**FIGURE 1:**
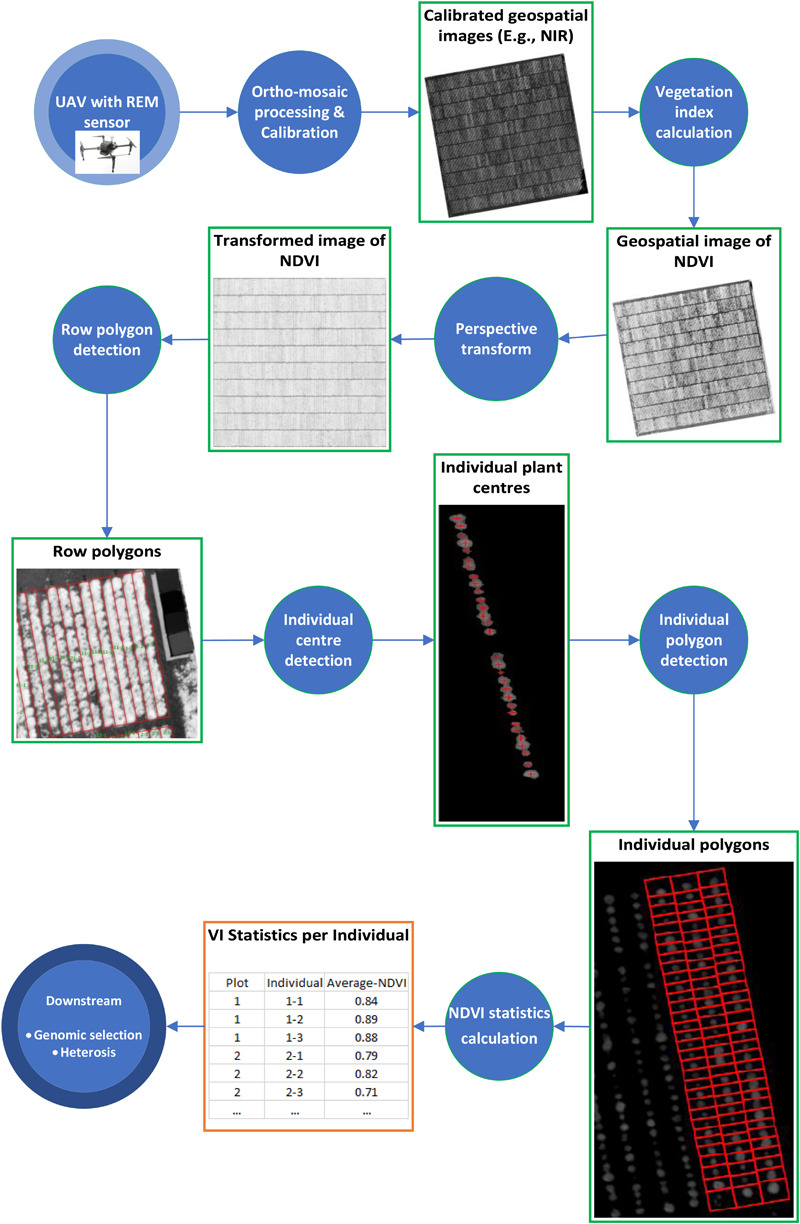
Workflow of analyzing geospatial images of spectral signals taken by REM sensor. Each circle represents a processing step and each rectangle represents an intermediate data type.

Before the first flight, nine ground control points (GCPs), (50 × 50 cm checkered linoleum flooring) were distributed across the field experiment to improve the georeferencing accuracy. The GCPs were used for manual assignment of absolute geographic coordinates to the ortho-mosaic images. For image processing, Pix4Dmapper Pro (version 4.3.31 Pix4D, Lausanne, Switzerland, https://pix4d.com) was used to process raw images into ortho-mosaics. The workflow of analyzing geospatial images of spectral signals (Figure 1) is described below.

#### Ortho-Mosaic Processing, Calibration, and NDVI Extraction

Ortho-mosaic processing have five main steps; including image alignment, image geo-referencing, building dense point clouds, ortho-mosaics, and vegetative indices map, were followed for image processing. For detailed processing steps, please refer to following previous studies (Khan et al., 2018; Dobbels and Lorenz, 2019). Ortho-mosaic images were generated and stored as geo-referenced TIFF files. Assessment accuracy of processed ortho-mosaic images was conducted by assessing the quality report from the Pix4D processing. After the ortho-mosaic processing was completed, reflectance calibration and NDVI extraction were conducted using eCognition. For radiometric calibrations, linear correlations were developed between ortho-mosaic images of DN and their known reflectance values for each band. The equation developed was used to convert DN to corrected reflectance values of the ortho-mosaic TIFF file. The corrected ortho-mosaic TIFF file was used to develop the NDVI extraction workflow in eCognition. NDVI was calculated from the red and near-infrared reflectance values (Khan et al., 2018).

We generated a polygon as the boundary of each row of 32 plants as well as a polygon around the boundary of each individual plant with an identification number (plant ID) from the calibrated NDVI images above. The process comprises of four components:

#### Perspective Transform

**FIGURE 2:**
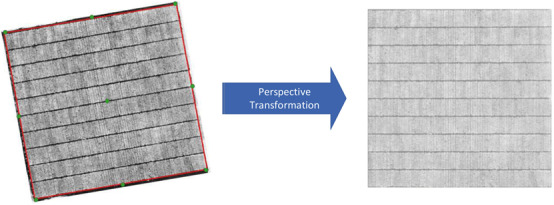
Transformation of GeoTIFF images based on 4 corner GCPs. The green points are GCPs and the red polygon is the boundary for the perspective transformation.

Four corner GCPs in the field were used to form a polygon bounding the area of interest. The area contained in this polygon for each NDVI image was subsequently cropped and rotated into a rectangular shape using a perspective transformation (Figure 2), where the plant rows and blocks were essentially orthogonal to the image axes. Identification of plant row polygons.

A set of 1,500 polygons, each of which contains a single row of plants, was generated based on each transformed NDVI image (time point). The best polygon for each plant row, which we refer to as a consensus polygon, was subsequently chosen and applied to all downstream analysis later.

**FIGURE 3:**
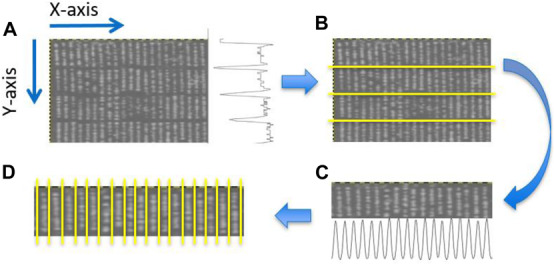
Identification of plant blocks and plant rows. **(A)** Partial view of an NDVI UAV field image. The trace to right of this image represents the sum of the pixel values along each row of the image producing a vertical profile. **(B)** Overlay of identified horizontal dividing lines separating the four plant blocks. **(C)** The projection method applied to each plant block was used to identifying the plant rows in each plant block. Here the horizontal profile seen at the bottom of **(C)** constitutes the sum of the pixels along each pixel column within the given block. **(D)** Overlay showing the separation obtained between adjacent plant rows for a particular block.

**FIGURE 4:**
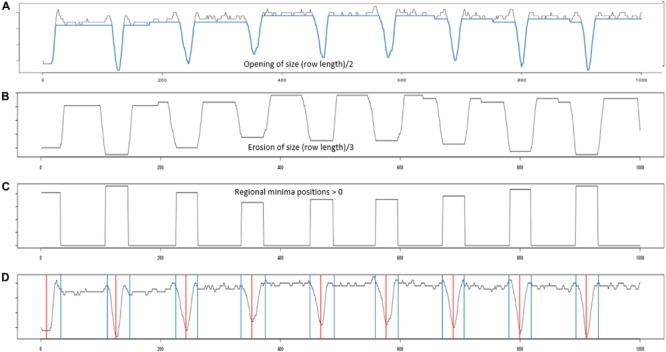
Profile processing. **(A)** Shows the projection profile from nine plant blocks. The blue overlay shows the result of performing a morphological opening using a line structuring element of length (plant row length)/2. **(B)** The opening was then processed using an erosion using a line structuring element of length (plant row length)/3. **(C)** Inverted regional minima. The eroded profile was processed to locate its regional minima. **(D)** Red vertical lines give the centers of each identified regional minima, while the blue vertical gives the boundary edge of each regional minima identified.

Plant rows (Figure 3D) for each transformed NDVI image were identified using projection methods, whereby the sum of the image pixels along the image columns and image rows was used to produce vertical and horizontal projection profiles. These profiles were then processed (Figure 4) to identify the centers of each trough (low-intensity regions) separating either the plant blocks (Figure 3B) or the plant rows (Figure 3D). Briefly: first, the plant blocks were identified by taking the projection of the image pixels values along the x-axis as shown in Figure 3A where the horizontal projection is seen in the profile to the right of the sub-image. The actual processing steps used to identify the troughs between the plant-blocks are laid out in Figure 4, from which it is seen that only one external parameter is required; that is the expected length of the trial plant rows. Next, once each plant block was isolated (Figure 3C) the individual plant rows were then identified using the same process outlined in Figure 4 in each plant block, but where the projection was along the y-axis, and the plant-row length parameter was replaced with the expected plant width/diameter.

**FIGURE 5:**
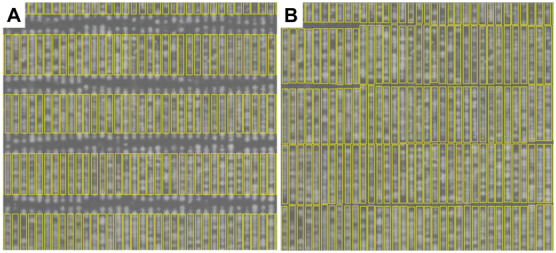
Plant-row box growing results. **(A)** Initial estimate of plan-row boxes. **(B)** The same plant-row boxes after box growing.

The edges associated with each plant-block and plant-row (Figure 4D, blue vertical lines) were then used to construct an initial estimate of each plant-row polygon/box (Figure 5A). As can be seen in Figure 5A, the initial estimates of the plant-row boxes underestimated the extent of each box in the vertical direction. The amount of underestimation in this case was deliberate and was controlled by the size of the erosion in the profile processing (Figure 4B). This was done to compensate for the lack of alignment in the actual start and end vertical position of each planted plot-row in each block (Figure 5A). However, this meant the trial plants between two vertically aligned plant-boxes were not assigned to a plant-row box. Therefore, to assign each of these plants to a plant-row-box a box growing procedure was developed as shown in Figure 5B.

In the box growing procedure, each initial plant-row box (Figure 5A) is linked with the boxes directly above and or below it. Each initial plant-row box is linked to at most two other boxes. The region between two linked boxes can be defined by a rectangle, with the top edge of the link delineated by the bottom edge of the top box, and the bottom edge of the link delineated by the top edge of the lower box. The sides of the rectangular linking region are defined by the minimum and maximum pixel x-positions taken from the intersection of the x-positions defined by the top and bottom boxes. Therefore, the width of a linked region is defined by the maximum x-position minus the minimum x-position plus 1.

Next, for each of the linking regions, a projection profile was constructed from the row-wise sums of the pixels in each of the rows in the linking region. Let such a profile be represented as f(x), where x∈{1,…,n} and n is the number of image pixel-rows between the linked boxes. f(1) represents the sum of pixels along the top edge of the link and f(n) represents the sum of the pixels along the bottom edge of the link. Let t represent a positive threshold value. The threshold t is set to the average background (non-plant) pixel intensity and is scaled during box growing to the number of pixels between the upper and lower edges of the growing boxes. This threshold can be determined from the image or can be supplied by the user. The details of the box growing procedure are outlined in the following pseudo-code:

**Table:** 

Let j = 1; k = n; a = 0; s = $\sum_{i=1}^{n}$f(i); t = specified threshold
width = number of pixels in a link row
while(j < k) {
if (f(j) > f(k))
a += f(j);
j++;
else if(f(j) == f(k))
a += f(j) + f(k);
j++; k–;
else
a += f(k);
k–;
t1 = t * width * (n-j+1);
if(s-a <= t1)
break;
}
Add to bottom edge of top box j-1 rows;
Add to top edge of bottom box n-k rows;

#### Choosing Consensus of Plant Row Polygons

As the plant row polygons were detected for each image separately, we chose a best set of polygons that could be applied to all downstream analysis. In order to choose the most stable set of polygons, the variance of the area of the 1,500 polygons for each transformed NDVI image (time point) was calculated and compared across all time points. The plant row polygons identified from the image captured at 31/03/2017 had the least variance in polygon area and were chosen as the consensus.

#### Individual Center Detection

To identify single plants within the GS field trial, we applied the following 2-step process.

Firstly, a center point of each individual plant was identified. The NDVI ortho-mosaic TIFF image (captured date: 31/03/2017), from which the consensus of plant row polygons was developed, and subsequently processed in Pix4D was imported into eCognition (eCognition developer 9.2, Trimble, Munich, Germany). The template editor algorithm in eCognition was used to allow for the identification of individual plants. A test template was created by selecting 35 samples (Width: 20; Height 20; Context: 4) which were representative of various sizes of perennial ryegrass plants. The correlation between the generated test template and selected samples was 0.774. A small-region of the NDVI layer was randomly selected to validate the generated template and predict individual plants within the area selected. The identified plants within template were then manually verified by the user as being correct, false, or unsure predictions to allow eCognition to refine the prediction model. After the template validation, test parameters values were obtained [ground truth tolerance (pixels): 3 and threshold: 0.6]. The template applied to the test region correctly identified 281 plants, missed 18 plants and incorrectly identified 2 samples as plants. The generated template was then updated based on the test area and the updated template, which had a sample correlation (0.917) to the samples within the selected area. This template matching algorithm was then applied to the trial area. All points predicted outside of the plant-row box was discarded. Manual visual quality control was performed within each plant-row box and further points added or removed to ensure that it was correct representative of the plants within the image were included.

#### Individual Polygon Detection

Once all plant-row-boxes were constructed, and all plant centers identified, a simple procedure was used to assign each plant-center to its enclosing plant-row-box. Once all plant-centers were assigned to a plant-row box, individual plant-boxes needed to be constructed around each plant center. This was done by (1) using the midpoint between adjacent plants-centers to create a horizontal line connecting the left and right edge within each plant-row-box and (2) the enclosing boxed region containing a plant-center was then identified and used to determine the coordinates for each plant-box. Consequently, each plant-row polygon was split into 32 individual plant polygons (bottom right Figure 1). Each individual plant polygon was also annotated by an identification number (plant ID) and was used to calculate the NDVI statistics for each plant in eCognition.

#### Ground-Based Plant Height Data Acquisition and Processing

In the ground-based platform, “PhenoRover,” a Polaris ranger side-by-side vehicle was used for ryegrass morphometric data collection. Details of the PhenoRover platform and data processing method was discussed in our previous paper (Gebremedhin et al., 2019a). In brief, the vehicle has six ultrasonic sonars for plant height measurement attached to a welded steel bar at 0.6 m above-ground. For accurate geolocating of each sensor’s data, a single Real-Time-Kinematic Global Navigation Satellite System (RTK-GNSS) unit was placed on the top of the vehicle. Sensors data was stored on a data logger (CR3000, Campbell Scientific, Inc., Logan, UT, United States). For extracting data, we first projected the latitude and longitude coordinates to Universal Transverse Mercator (UTM) coordinate system and calculated the sensor position followed by matching sensor data to individual plants in the quantitative GIS software interface. The same polygons used to extract NDVI were superimposed on top of each geo-referenced data in the quantitative GIS software interface to extract plant height.

### Destructive Biomass Measurements

The aboveground biomass was harvested manually at 5 cm height. Destructive biomass harvest of 480 space-plants was collected following the same procedure described previously (Gebremedhin et al., 2019a). In addition, mechanical cutting of all the GS field experiment plots was conducted to measure total fresh biomass weight (kg/plot) at the row or plot level. In this case, biomass was measured at each seasonal harvest from all 500 plots. This data was used to validate the computational phenotyping workflow with manually derived polygons as well as to compare manually collected biomass with predicted biomass using the workflow.

### Data Analysis

The variability distribution of phenotyping data was statistically analyzed using R version 3.6.1, R Development Core Team, Vienna, Austria. Non-destructive biomass estimation of individual plants was described using the combination of NDVI and plant height. Spatial distribution variance maps of NDVI, plant height, predicted and measured DMY in three seasons were compared. Histogram of distribution graphs was prepared in Tableau Software (Tableau2019.4, Seattle, Washington, United States). The predicted DMY of spaced-planted individuals was therefore estimated using the NDVI and height combination (NDVIsq_PH) using the following general and seasonal formulas derived from Gebremedhin et al. (2019a):

#### Universal Equation:

(1) Biomass = intercept+NDVIsq_PH+Season+NDVIsq_PH×Season

#### Seasonal Equations:

(2) winter2018=-4.62+NDVIsq⁢_⁢PH

(3) late-spring2018⁢_⁢1=-4.62+(5.088×NDVIsq⁢_⁢PH)+5.76+(1.49×NDVIsq⁢_⁢PH)

(4) late-spring2018⁢_⁢2=-4.62+(5.088×NDVIsq⁢_⁢PH)+16.86+(3.68×NDVIsq⁢_⁢PH)

The statistical regression between the predicted and measured DMY during each harvest was compared at single plant and plot level. Similarly, a model considering all three harvests of biomass estimation was also developed. Coefficient of determination (*R*^2^) and root mean square error (RMSE) were used to evaluate and quantify the accuracies of regression models of phenotyping variables. Normality of the data was verified by checking the residual vs. fitted values of plots to ensure the normality of residuals with constant variance.

## Results

### Comparison of Automated and Manually Derived Delineated Polygons

**FIGURE 6:**
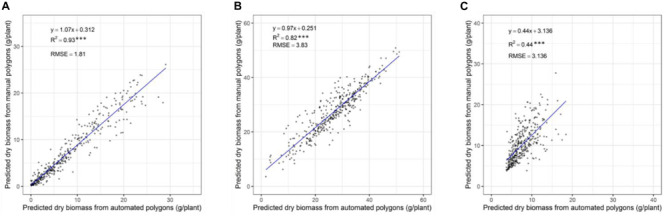
Comparison of predicted DM of individual plants through automatically and manually derived polygons across growing seasons of winter2018 **(A)**, late spring2018_1 **(B)**, and late spring2018_2 **(C)**.

In our previous validation experiment, we developed a model to predict the DMY of individually drawn polygon by combining NDVI and plant height (Gebremedhin et al., 2019a). Polygons used to extract data were manually, but it is difficult to apply this into 48,000 plants. As a result, a computational phenotyping workflow was applied to extract NDVI and plant height information. To evaluate the robustness of the automatically derived polygons, the predicted DMY of individual plants from automated and manually derived polygons was compared across three different seasons (Figures 6A–C). The results show a strong coefficient of determination between predicted DMY from automated and manual polygons with *R*^2^ values ranging 0.82-0.93 for the winter2018 and late-spring2018_1 season and RMSE values ranging from 1.81 to 3.83 g/plant. For the late-spring2018_2, harvest a moderate coefficient of determination (*R*^2^ = 0.44) of DMY prediction was obtained. These results indicate that automatically built polygons can replace manually derived polygons for plant height and NDVI accurate extraction, and biomass prediction for all 48,000 plants in our field experiment.

### Seasonal Spatial Variation of Biomass and Other Traits

**FIGURE 7:**
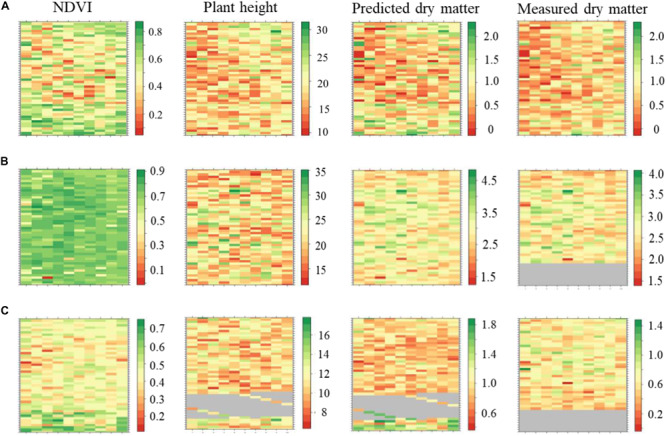
Maps of perennial ryegrass NDVI, plant height (cm), predicted and measured DM (kg/plot) spatial variations at Winter2018 **(A)**, late Spring2018_1 **(B)**, and late Spring2018_2 **(C)** at harvest time.

The plant height, NDVI and predicted DMY variables were retrieved using a computational data processing workflow developed in this experiment (Figures 7A–C). NDVI showed seasonal variability with increased values observed in late-spring2018_1 compared to winter2018 and late-spring2018_2. Most of the plots in the late-spring2018_1 indicated NDVI was at saturation point at the time of harvest. Spatial variations of NDVI values were observed in the middle, top and lower part of the field experiment in all seasons. For instance, the center part of the GS trial site showed explicitly lower NDVI values in winter2018 compared to an upper and lower location within parts of the field experiment. Seasonal plant height and predicted biomass variation occurred in the winter2018 followed by peak accumulation in the late-spring2018_1 then decreased into late-spring2018_2.

### Distribution of NDVI, Plant Height, and Predicted Biomass Across Growing Seasons

**FIGURE 8:**
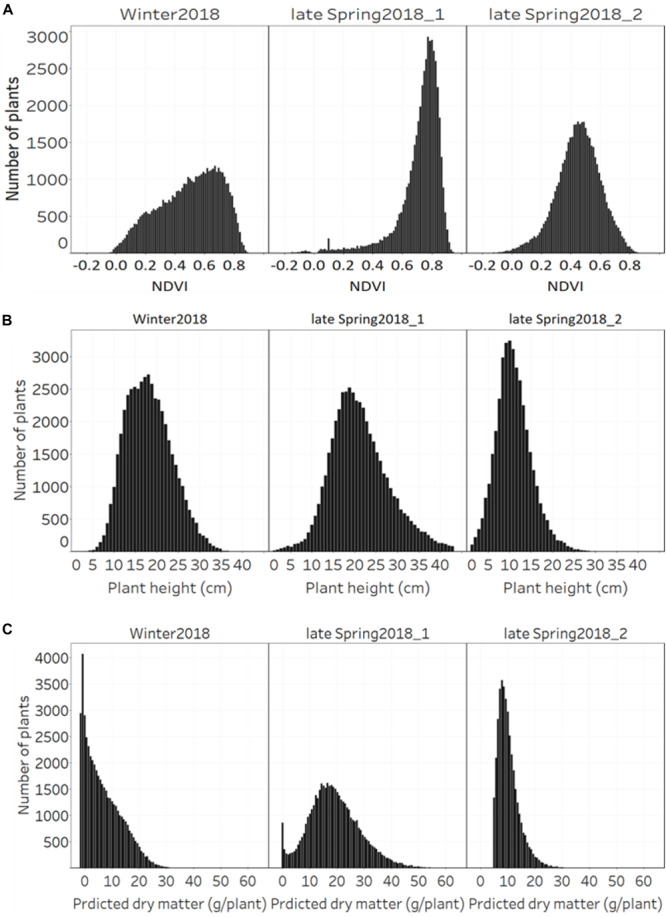
Distribution of **(A)** NDVI, **(B)** plant height, and **(C)** predicted DM across three growing seasons of 48,000 individual perennial ryegrass genotypes.

The distribution of NDVI, plant height and predicted biomass across three growing seasons, of the entire population of 48,000 individual perennial ryegrass genotypes was estimated (Figures 8A–C). Figure 8A shows the NDVI distribution variation of individual plants across seasons. The winter2018 season had a wider spread of NDVI values at the time of harvest for most genotypes, with some already reaching saturated NDVI values. In the late-spring2018_1, the narrow spread of NDVI values distribution was skewed toward the right indicating that most of the individual plants reached the NDVI saturation point at the time of harvest. In the late-spring2018_2 harvest, relatively normalized distribution of NDVI values was observed signifying some individual plants were harvested earlier than the 2–3 leaf stage.

The plant height of spaced-plants showed a normal distribution across all growing seasons (Figure 8B). Taller plants were observed at the late-spring2018_1 harvest followed by the winter2018 and late-spring2018_2 harvests, respectively. Figure 8C shows the predicted DMY distribution variation of individual plants across seasons. The winter2018 and late-spring2018_2 seasons histogram distributions were similar with left-skewed distributions of narrow-spread values at the time of harvest for most genotypes. The predicted DMY distribution of the late-spring2018_1 harvest was observed to be normally distributed, contrary to the winter2018 and late-spring2018_2 harvests.

### Comparison of Seasonal Distribution of NDVI, Plant Height, and Predicted Biomass of Cultivars

**FIGURE 9:**
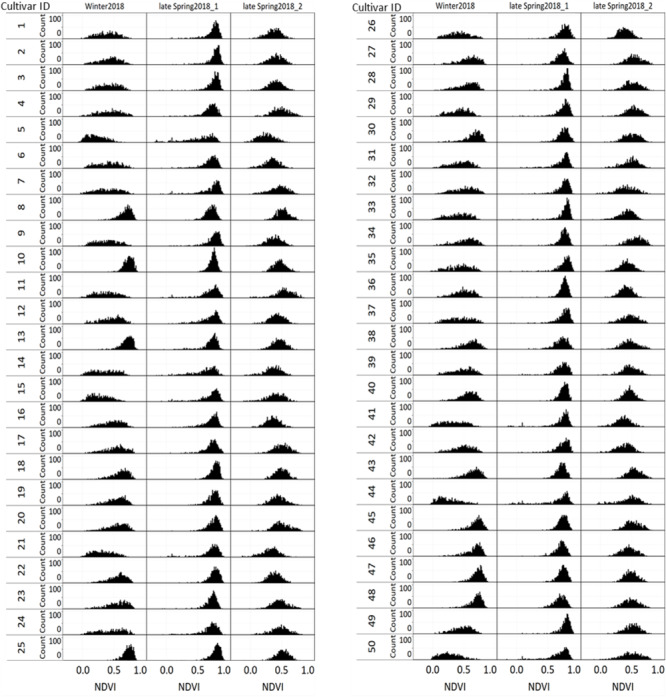
Variation of individual plants NDVI values among and within 50 perennial ryegrass cultivars/advanced breeding lines across three growing seasons.

Figure 9 displays the NDVI histogram distribution variance of 50 cultivars/breeding lines for each of the three harvests. Cultivars showed large relative differences in growth and development between and within a harvest. For example, cultivars designated with a Cultivar ID number 8, 10, 13, 25, 45, 46, 47, and 48 reached saturation and the overdue harvest was observed in winter2018.

**FIGURE 10:**
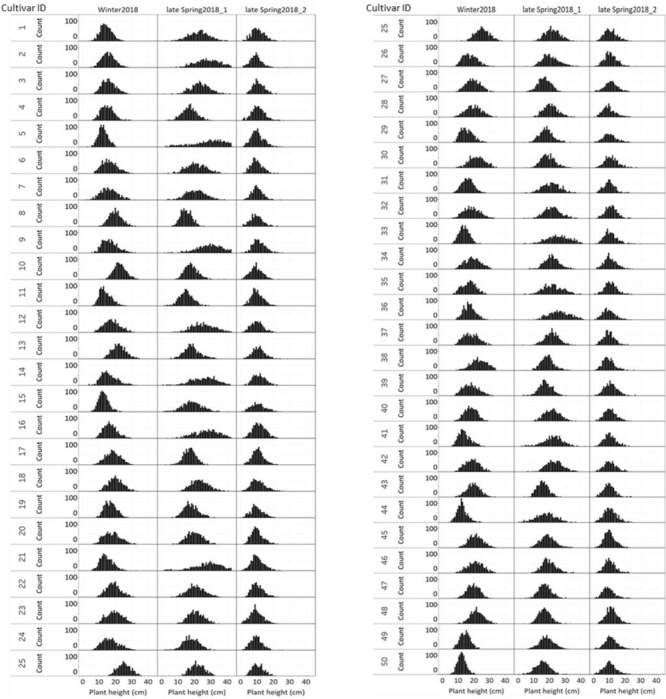
Variation of individual plants height among and within 50 perennial ryegrass cultivars/advanced breeding lines across three growing seasons.

Figure 10 displays the plant height variation distribution of 50 cultivars/breeding lines across three seasons. Similar to the NDVI histogram distribution, the plant height variation of each cultivar was obvious at the winter2018 harvest. For instance, cultivars designated with Cultivar ID number 8, 10, 13, 25, 45, 46, 47, and 48 showed higher height values than their counterparts. Similarly, plants with Cultivar ID 5, 9 12, 14, 16, 21, 33, 35, 36, and 41 showed a wider distribution at the late-spring2018_1 harvest. The plant height distribution for the late-spring2018_2 harvest did not show any major histogram structural differences among cultivars.

**FIGURE 11:**
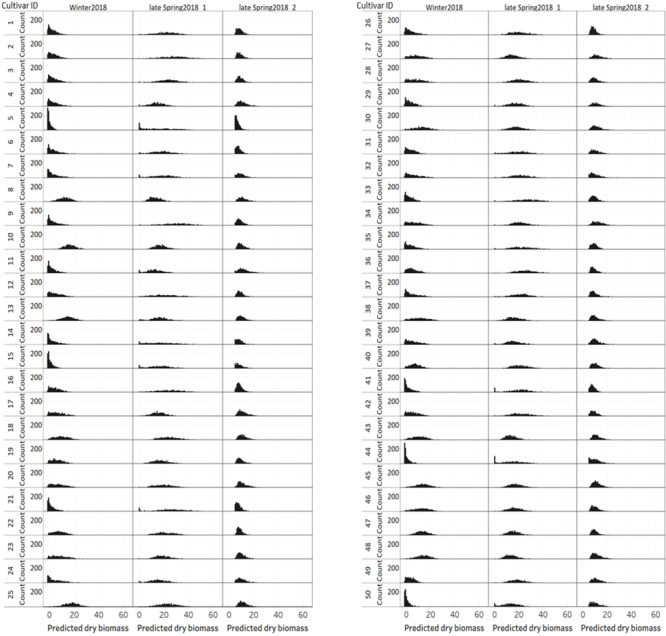
Variation of individual plants biomass (predicted) among and within 50 perennial ryegrass cultivars/advanced breeding lines across three growing seasons.

Figure 11 displays the variation of individual plant biomass (predicted) among and within 50 perennial ryegrass cultivars/advanced breeding lines across three growing seasons. Similar to the plant height and NDVI, cultivars designated with a Cultivar ID number 8, 10, 13, 25, 45, 46, 47, and 48 showed higher height values. However, the histogram of predicted DMY for each cultivar at the late-spring2018_1 harvest showed more variation compared to the winter2018 and late-spring2018_2 harvests. Moreover, varietal differences in DMY distribution of some cultivars showed a clear difference at the winter2018 harvest.

### Plot Level Seasonal Biomass Estimation Model

**FIGURE 12:**
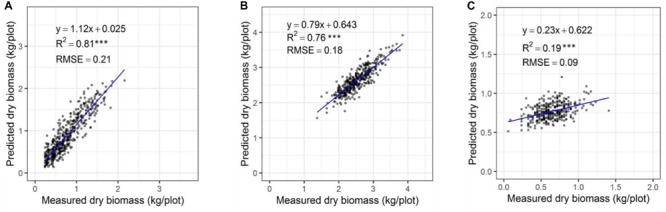
Comparison of predicted and measured DM (kg/plot) based on spaced-planted trial in Winter2018 **(A)**, Late Spring2018_1 **(B)**, and Late Spring2018_2 **(C)**.

**FIGURE 13:**
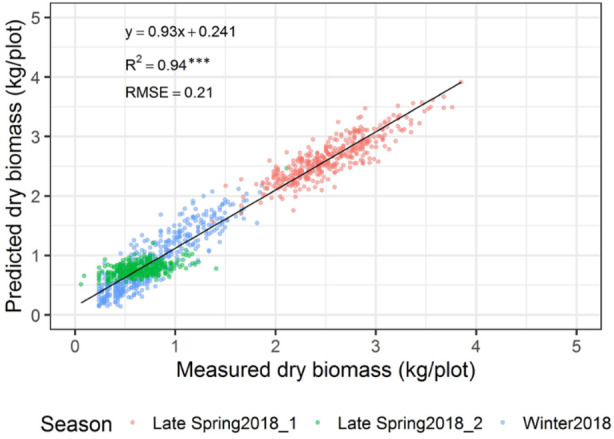
Comparison of predicted and measured DM (kg/plot) across three growing seasons combined.

The automated biomass prediction of spaced individual plants was translated to plot-level estimates. The sum of predicted biomass from spaced planted 96 individual plants within the plot was calculated to determine predicted biomass per plot and compared with the mechanically harvested and measured biomass data. Significantly high coefficients of determinations were observed between measured and predicted DMY (*R*^2^ = 0.19-0.81) at all three harvests (Figures 12A–C). Subsequently, RMSE values ranged from 0.09 to 0.21 kg/plot was observed at these harvests. When combining all harvests into a single regression of estimated and measured biomass (Figure 13), the best coefficients of determination had a slope, *R*^2^ and RMSE values of 0.93, 0.94, and 0.21 kg/plot, respectively.

## Discussion

In commercial ryegrass breeding, experiments with thousands of individual space-plants may be used for evaluation and selection (Hayes et al., 2013; Lin et al., 2016). Phenotyping of these genotypes requires repeated evaluation across growing seasons and years. This is essential to select, potentially high yielding genotypes to take into future stages of a breeding program. However, phenotyping of large populations, by the available traditional methods of visual scores, manual cutting, bagging and drying is difficult to implement in terms of time and cost. This study developed and validated ground-and aerial-based platforms equipped with advanced sensors for high-throughput phenotyping of ryegrass biomass yield. This resulted in the development of a computational workflow for image acquisition, processing, and analysis to predict biomass yield based on vegetative indices and plant height from 48,000 ryegrass plants. With this method, it was possible to assess traits including predicted biomass of 48,000 individual plants in 6 h from data acquisition to data extraction. This method may provide a robust method for ranking of genotypes and make faster progress in breeding programs.

Manually drawing thousands of polygons has been indicated difficult and considered as time-consuming and automation of polygons for 38,000 plots was expected to increase the throughput and accuracy of traits extraction (Shi et al., 2016). A coefficient of determination between the predicted DMY from automated and manually derived polygons in the late-spring2018_2 was moderate (*R*^2^ = 0.44). This could be due to smaller plant height architecture at that particular time, which coincided with early drought onset in the spring season of 2018. The canopy of these smaller plants may have reflected weak sound echoes from the surface of each plant, which may result in a greater variability in height values that resulted lower *R*^2^.

Biomass yield distribution of perennial ryegrass cultivars may vary across growing seasons and years (Brock et al., 1996; Chapman et al., 2015). These variations mainly occur due to genetic merit of productivity and seasonal growth rate differences of cultivars during the vegetative growth stage. The ability to measure how much differences exist mainly depends on destructive harvest or visual score, which is restricted by low-throughput (Walter et al., 2012; Ghamkhar et al., 2019). Sensor-based non-destructive biomass yield predictive methods have the potential to replace this with applicability in large scale screening of breeding lines across seasons and years. Generally, individual plants NDVI, plant height and predicted biomass estimations showed variations across seasons. The distribution of NDVI values was wider in the winter2018 than late spring2018_1 and late spring2018_2. This indicates the various genotypes responded differently to cold season conditions (Förster et al., 2018). However, plant height and DMY predictions in the late spring2018_1 indicated higher values in which ryegrass appeared to have rapid growth and faster accumulation of dry matter in the spring season (Wang et al., 2019).

In this study, NDVI, plant height and predicted biomass estimations from the UAS and PhenoRover showed obvious temporal and spatial variabilities that mainly arises from genetic and environmental interactions. In the winter2018 harvest, NDVI histograms of some cultivars (Cultivar ID 8, 10, 13, 25, 45, 46, 47, and 48) indicated they were at NDVI saturation point before the harvest. This was further confirmed by the estimates of the plant height and DMY of these cultivars. These methods of accurate non-destructive estimates may provide a better perspective to facilitate breeding selection and ranking of ryegrass genotypes. The application of this non-destructive biomass prediction will enable the development of seasonal forage value indices (FVI) which combine biomass yield and economic values to select best performing commercial cultivars (Leddin et al., 2018; Giri et al., 2019). At the late-spring2018_1 harvest, plant height increased, and NDVI values saturated for all cultivars. The increase in plant height was from the fast growth rate in the spring season and NDVI value peaked and reached saturation before harvest. This result agrees with previous studies in wheat (Li et al., 2019) and buffelgrass (Mutanga and Skidmore, 2004) where NDVI tended to saturate at high canopy density. However, in the late-spring2018_2 lower NDVI, plant height and predicted biomass yields were observed for some plants. This indicates there was an early-onset of a late spring drought stress, that can restrict leaf growth (Cyriac et al., 2018), and plants with a thin canopy density are expected to reflect limited echoes and NDVI values (Condorelli et al., 2018; Yuan et al., 2018).

Experimental plots in this study were represented by three sets of rows containing 32 spaced-planted individuals each (total 96 plants/plot), in which individual plants level biomass prediction has reflected an accurate representation of plots biomass prediction. The results showed a significantly higher coefficients of determination between the predicted and measured DMY across three seasons with *R*^2^ = 0.19, 0.75, and 0.81 at the winter2018, late-spring2018_1 and late-spring2018_2 harvests, respectively, and RMSE values ranging from 0.09 to 0.21 kg/plot. Subsequently, the overall regression of the three harvests indicated significantly high coefficients of determination (*R*^2^ = 0.94) and RMSE 0.21 kg/plant. Similar coefficients of determination trends (*R*^2^ = 0.76-0.89) were observed in previously reported pasture studies when various sensors were used to estimate biomass yield of tall fescue and ryegrass (Safari et al., 2016; Ghamkhar et al., 2019).

In this study, we validated a computational workflow for image acquisition, processing, and analysis to predict the biomass yield based on vegetative indices and plant height measurements of 48,000 ryegrass plants. Previous studies indicated the use of NDVI for ranking cultivars of ryegrass (Wang et al., 2019), field pea, canola, and spring wheat grain yield (Brian McConkey et al., 2004) and lint yield in cotton (Hugie et al., 2018). Considering the plant height and NDVI as a surrogate to predict DMY of individual and plot-level plants, there is a great potential to apply our workflow to be used for ranking of genotypes and cultivars across growing seasons and years. Our method also has the potential to further pave a foundation for the application of sensor technologies combined with GS that leads to faster rates of genetic gain in forages. To further improve the stability and accuracy of the automated workflow, other traits including vegetative indices other than NDVI, volume and leaf area features may be included by incorporating measurements through sensors including LiDAR and various camera systems (Ghamkhar et al., 2019; Yates et al., 2019).

## Conclusion

The computational phenotyping workflow developed in this study indicated that automatically built polygons could replace manually derived polygons for plant height and NDVI accurate extraction from 48,000 plants. The NDVI, plant height, predicted and measured DMY showed accurate seasonal and spatial variations, which may contribute to identifying differences among genotypes of varying genetic background. Thus, the proposed computational phenotyping workflow proved to enable non-destructive and objective prediction of individual plants NDVI, plant height and DMY in three different seasons. Subsequently, the observed plot level linear relationship between predicted and measured DMY indicated capability of the developed computational workflow to estimate plot-level DMY from the individual plants’ prediction. Therefore, this study demonstrated the potential of the validated computational workflow to conduct seasonal and annual ranking of breeding lines and commercial cultivars that may be used for the application of genomic selection that paves to greater rates of genetic gain in forages.

## Data Availability Statement

All datasets generated for this study are included in the article/Supplementary Material.

## Author Contributions

KS, PB, JW, and GS conceived the research. KS, PB, and JW designed and participated in charge of overall supervision of the study. KS and AG drafted the manuscript. AG wrote the manuscript with input from all authors. AG and KG analyzed the data. PB and AG analyzed images and plant height data. EB, PB, and FS developed the automated polygons used to segment images and plant height data from the spaced plant trial.

## Conflict of Interest

The authors declare that the research was conducted in the absence of any commercial or financial relationships that could be construed as a potential conflict of interest.

## Supplementary Material

FIGURE S1 Experimental field site: A genomic sub-selection (GSS) experiment on perennial ryegrass at Hamilton Centre Research Station in Victoria State of Australia was conducted in three growing seasons in 2018. RGB ortho-mosaic image is used to display the GSS field experiment site, ground control points, and delineated polygons.
