# Supplementary figures and images for: Development and Validation of a Phenotyping Computational Workflow to Predict the Biomass Yield of a Large Perennial Ryegrass Breeding Field Trial
Source: Front Plant Sci. 2020 May 28;11:689. doi: 10.3389/fpls.2020.00689 (PMC7270830; doi:10.3389/fpls.2020.00689)

Supplementary Figure 1.


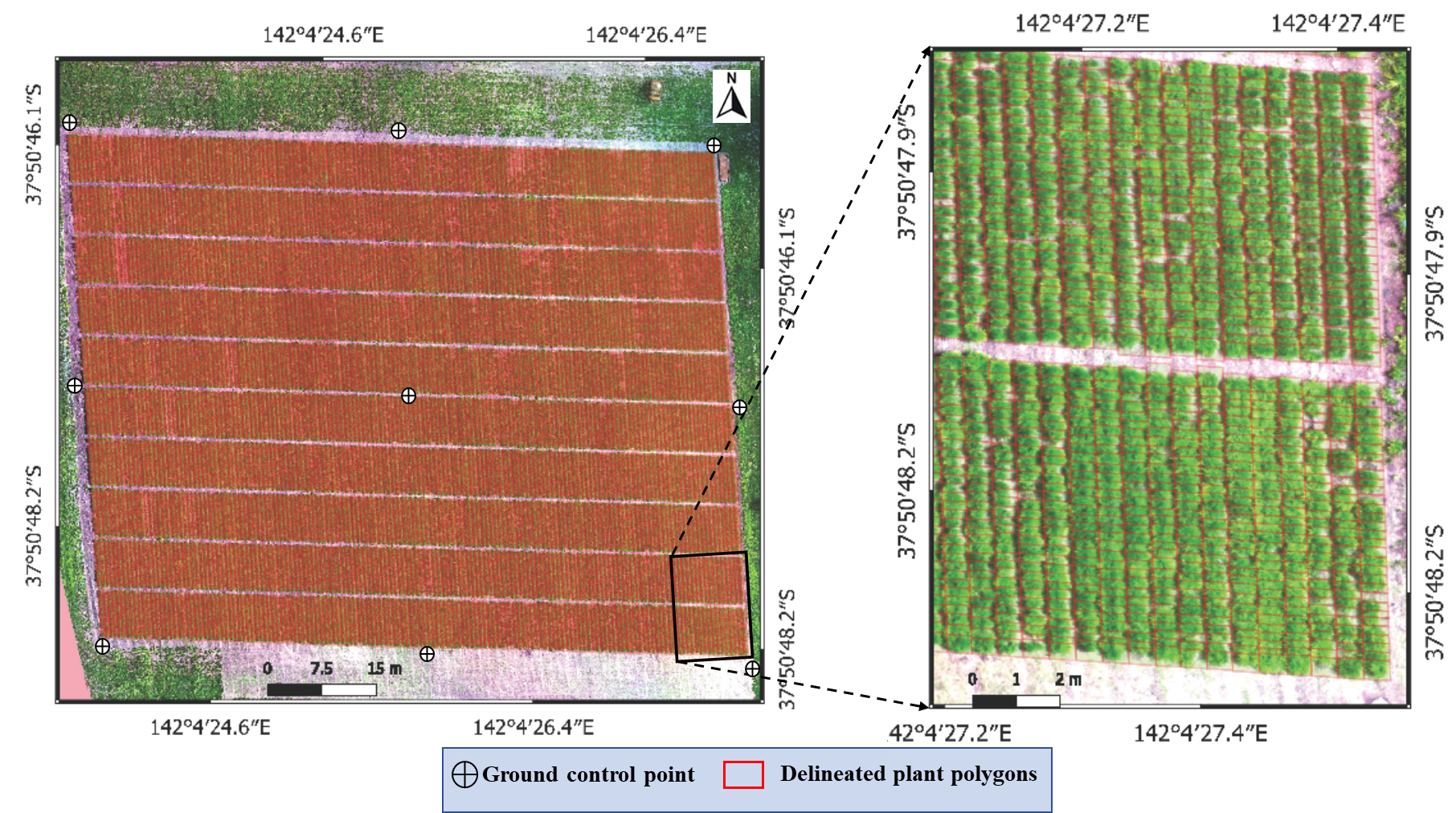

Supplement: FIGURE S1 — Experimental field site: A genomic sub-selection (GSS) experiment on perennial ryegrass at Hamilton Centre Research Station in Victoria State of Australia was conducted in three growing seasons in 2018. RGB ortho-mosaic image is used to display the GSS field experiment site, ground control points, and delineated polygons. [file Data_Sheet_1.docx]
